# Hypertension linked to Alzheimer’s disease via stroke: Mendelian randomization

**DOI:** 10.1038/s41598-023-49087-0

**Published:** 2023-12-07

**Authors:** Chao Tang, Yayu Ma, Xiaoyang Lei, Yaqi Ding, Sushuang Yang, Dian He

**Affiliations:** Department of Neurology, Affiliated Hospital of Guizhou Medical University, No. 28, Guiyi Street, Yunyan District, Guiyang, 550004 Guizhou Province China

**Keywords:** Cognitive ageing, Cognitive neuroscience

## Abstract

This study aimed to investigate the relationship between hypertension and Alzheimer’s disease (AD) and demonstrate the key role of stroke in this relationship using mediating Mendelian randomization. AD, a neurodegenerative disease characterized by memory loss, cognitive impairment, and behavioral abnormalities, severely affects the quality of life of patients. Hypertension is an important risk factor for AD. However, the precise mechanism underlying this relationship is unclear. To investigate the relationship between hypertension and AD, we used a mediated Mendelian randomization method and screened for mediating variables between hypertension and AD by setting instrumental variables. The results of the mediated analysis showed that stroke, as a mediating variable, plays an important role in the causal relationship between hypertension and AD. Specifically, the mediated indirect effect value for stroke obtained using multivariate mediated MR analysis was 54.9%. This implies that approximately 55% of the risk of AD owing to hypertension can be attributed to stroke. The results suggest that the increased risk of AD owing to hypertension is mediated through stroke. The finding not only sheds light on the relationship between hypertension and AD but also indicates novel methods for the prevention and treatment of AD. By identifying the critical role of stroke in the link between hypertension and AD, this study provides insights into potential interventions that could mitigate the impact of hypertension on AD. This could help develop personalized treatments and help improve the quality of life of patients with AD who suffer from hypertension.

## Introduction

Alzheimer’s disease (AD), the most common neurodegenerative disorder, is characterized by memory impairment, cognitive deficits, and behavioral abnormalities caused by pathological changes such as β-amyloid (Aβ) deposition, formation of neurofibrillary tangles with hyperphosphorylated tau protein, and uncontrolled microglia activation in the brain^1,2^. With social development and population aging, the number of patients with AD in China is increasing rapidly, and the condition poses a significant public health challenge, with approximately 7 million people affected and an incidence rate of approximately 6.25/1000 person-years^3,4^.

Stroke, another major neurological disorder, causes different types of disability in approximately 90% of survivors^5^ and is a prominent cause of death in people with disabilities^6,7^. Ischemic stroke resulting from arterial occlusion owing to cerebrovascular obstruction is the primary causative factor in such disabilities, accounting for approximately 85% of all stroke cases^8^. Ischemic stroke comprises subtypes such as ischemic large vessel atherosclerotic thrombosis, cardiogenic embolism, and small vessel disease^9,10^. Increasing evidence indicates a reciprocal relationship between stroke and AD, which often manifest together and influence each other. A systematic evaluation and meta-analysis investigating this association reported an independent and significant increase in AD risk owing to stroke^11^. Conversely, another study showed that the incidence of stroke was 67% higher in patients with AD than in controls, primarily owing to higher rates of intracerebral hemorrhage (ICH). However, no significant difference was observed in the incidence of ischemic stroke between patients with AD and control patients^12^. These conflicting findings suggest that the relationship between stroke and AD is unclear and necessitates more extensive research.

Recently, hypertension was found to be strongly associated with both Alzheimer's disease (AD) and stroke, and chronic hypertension is considered one of the most prominent modifiable risk factors for AD. Epidemiological studies have shown that at least 25% of adults and over 50% of people aged above 60 years suffer from hypertension^13–15^. Results from imaging studies further support the link between hypertension and AD^16,17^. White matter lesions were found to be more pronounced in hypertensive patients than in normotensive individuals; this is one of the early pathological characteristics of AD. Similarly, a decrease in gray matter volume in hypertensive patients compared to that in normotensive patients was found to be associated with AD development and progression. This mechanism may be driven by various cerebrovascular lesions, such as atherosclerosis, microangiopathy, and cerebral hemorrhage, which are more severe in hypertensive patients^18,19^. These lesions can affect the normal function of cerebral blood vessels. Cerebrovascular lesions impede blood supply to the brain, thereby damaging the function of brain cells and neurons. These injuries may eventually cause AD^20^. In addition, hypertension may increase the risk of AD by impeding the circulation and metabolic processes in the brain. Hypertension can cause abnormal cerebral blood vessel constriction and dilation, thereby affecting the blood supply to the brain^21^. Insufficient blood supply to the brain may lead to hypoxia and cause malnutrition in brain cells, thereby increasing the risk of AD^20^.

In a recent study, genetic variants related to AD caused by hypertension were identified. Researchers analyzed 37 shared genetic loci between AD and hypertension through genome-wide association studies (GWAS), including coding genes and non-coding regions, some of which were previously associated with AD. Further functional enrichment analysis revealed that these loci involve multiple pathways related to AD^22^. Additionally, the study provided preliminary information on the mechanism by which hypertension causes AD. The findings showed that hypertension may be associated with calcium ion homeostasis disorder and the regulation of PKA^23^. Hypertension may affect the onset of AD by increasing intracellular calcium ion concentration and PKA activity^24^. This suggests that the two diseases may have some genetic overlap as well as related pathogenic mechanisms. In another study, the strongest genetic risk factor for AD, APOE ε4, was found to potentially modulate the association between hypertension and cognitive decline. APOE ε4 carriers induced more severe cognitive decline among individuals with hypertension. This suggests that the APOE ε4 gene may exacerbate the impact of hypertension on cognitive function. The mechanisms by which hypertension and APOE ε4 gene collectively contribute to cognitive decline remain unclear. One potential explanation is that hypertension and APOE ε4 gene expression may affect brain function and structure through different pathways. Hypertension leads to cerebrovascular disease and brain damage, whereas APOE ε4 gene expression may cause neuronal damage and inflammatory responses. These changes may be related and result in cognitive decline^25^. Clinical and molecular mechanisms both indicate that hypertension plays an important role in the process of AD induction.

Hypertension is also considered one of the major risk factors for stroke and plays a crucial role in stroke morbidity and mortality. The prevalence of hypertension has been reported to range from 30 to 80% in stroke patients and accounts for 32% to 45% of population risk factors of stroke^26^. Hypertension-induced cerebrovascular lesions, including cerebral atherosclerosis and microvascular lesions, can lead to cerebrovascular obstruction and cerebral hemorrhage, which cause stroke. Notably, hypertension also leads to heart disease, such as myocardial infarction and heart failure, which increase the likelihood of stroke^27^.

In summary, hypertension is widely acknowledged as a significant risk factor for both AD and stroke, with stroke being a risk factor for AD. In this study, we used Mendelian randomization (MR) to further investigate this relationship.

Mendelian Randomization (MR) is a powerful analytical method that uses genetic variants, specifically single nucleotide polymorphisms (SNPs), as instrumental variables (IVs). The random assignment of human genes in MR helps conduct a controlled test and reduces confounding factors, thus enabling the estimation of causal effects in the presence of unobserved confounding and measurement errors in exposure^28^. Thus, MR results are more robust and stable^29^. MR has gained increasing popularity in recent years in the investigation of exposure-outcome associations. A novel MR research method, mediated MR, uses a randomized trial design in which genotype is a mediating variable used to determine whether a biological indicator or disease mediates the relationship between another causal variable and a disease. Mediated MR is based on the fundamental idea of MR, which infers the relationship between a biological indicator or disease and another causal variable using the relationship between a genotype and the given biological indicator or disease. In this study, we used mediated MR to demonstrate the critical role of hypertension in the process leading to AD via stroke.

## Materials and methods

### Study design

**Figure 1:**
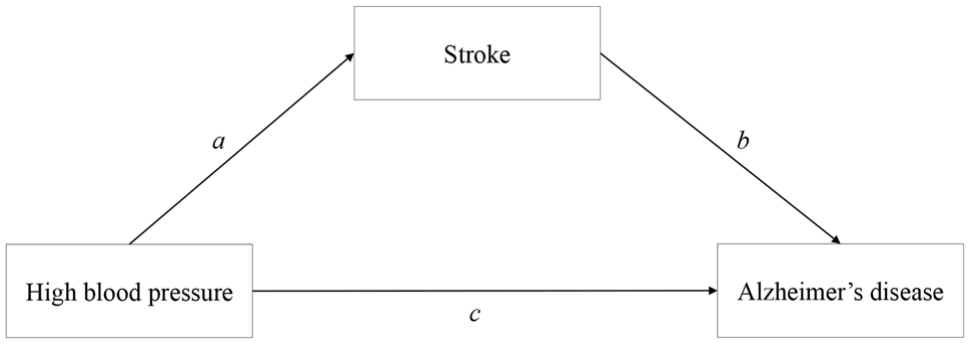
Use the two-step mesomeric effect (where a is the total effect of hypertension on stroke, and b is the effect of stroke on AD) and the product method (a × b) Direct effect = c−mesomeric effect.

In this study, we primarily used a two-sample and mediation MR method. Hypertension was considered as the exposure factor, AD as the outcome, and stroke as a mediator variable. The process is illustrated in Fig. 1, which shows the relevant flowchart. We used data from GWAS, in which valid instrumental variables (IVs) were selected and subjected to statistical analysis. MR analyses were performed using methods such as IVW, weighted median, MR-Egger, simple mode, and weighted mode. To validate the reliability of the results, we conducted sensitivity analyses, including MR-Egger regression and leave-one-out analysis, to assess horizontal pleiotropy and the presence of horizontal pleiotropic effects^30^. Additionally, our study met the three assumptions of MR: (1) association assumption: the genetic variation/instrumental variables (IVs) should be strongly correlated with the exposure factor. (2) independence assumption: the genetic variation/instrumental variables should be independent of any confounding factors that influence the exposure factor and the outcome. (3) exclusion restriction assumption: the genetic variation/instrumental variables should only affect the outcome through the exposure factor and not via other pathways^31^.

### GWAS data sources

To conduct two-sample MR analysis, we obtained summary statistics data from GWAS from the MR-Base platform (http://gwas-api.mrcieu.ac.uk/). To ensure data consistency, all data were sourced from individuals of European ancestry. In this study, we obtained exposed GWAS data from the UK Biobank, which included 9,851,867 SNPs from 3,646,613 samples. Our exposure ID on the openGWAS website is ukb-b-8746. For the mediator variable GWAS data, we used multi-ethnic GWAS that included 520,000 participants. In this study, the authors identified 32 loci associated with stroke and stroke subtypes^32^. We used 440,328 samples and 7,535,779 SNPs from this study. Our mediator ID on the openGWAS website is ebi-a-GCST005843. The outcome measures were obtained from the FinnGen database, which is an open resource from which researchers can access data by visiting the website and applying for access. The FinnGen database also provides a range of tools and resources to aid researchers in data analysis and interpretation. Our outcome ID on the openGWAS website is finn-b-G6_AD_WIDE_EXMORE.

### Tool variable selection

Instrumental variables (IVs) were selected based on genome-wide significance with a P value less than 5e−08 for SNP-gene correlation. We used the TwoSampleMR R package to filter SNPs in linkage disequilibrium (LD) using a threshold of r^2^ = 0.001 and kb = 10,000 for SNP selection.

### Statistical methods

In this study, we used five different MR models, namely IVW, MR-Egger regression, weighted median, simple mode, and weighted mode, to investigate the causal relationships among hypertension, stroke, and AD using two-sample MR analysis. IVW is the most commonly used model, being a weighted mean analysis of individual SNP results. However, since the IVW model assumes that all SNPs are valid instruments and may have directional pleiotropy, the estimates could be biased^33^. Therefore, we performed sensitivity analyses using different methods to reduce the bias in our results. We used R software's heterogeneity function to test for heterogeneity in instrumental variables. If p < 0.05, it indicates that the instrumental variables are different from each other. In the presence of heterogeneity, we use a random effects model for analysis, which is meant for handling heterogeneity. In this model, the effect of each genetic variation is considered as a random variable rather than a fixed value. This allows for the consideration of heterogeneity between different genetic variations and assigns different weights to the estimated values of different genetic variations. By using the random effects model, causal effects can be estimated more accurately, while taking into account the influence of heterogeneity, In the presence of pleiotropy, we conducted further sensitivity analyses to assess the impact of pleiotropy on the results. Sensitivity analyses can help evaluate the degree to which pleiotropy affects the results. By conducting sensitivity analyses in pleiotropy, we can determine the sensitivity of the results to pleiotropy and assess the stability of the interpretation of the results in the presence of pleiotropy. We also used the pleiotropy function to perform multiplicative tests, with the MR Egger method represented by the intercept term. When p < 0.05, multiple instruments were identified. Additionally, we performed a leave-one-out sensitivity test to reassess data quality, and we presented the results of relevant sensitivity analyses using forest plots and leave-one-out plots.

In the mediated Mendelian randomization study, we used the two-step mediated MR method, which is used for assessing causality and mediating effects. This approach helps researchers accurately assess causality and identify mediating factors, thereby providing better guidance for disease prevention and treatment^34^. Multiple steps were performed to ensure the reliability and validity of our analysis, including instrument variable selection, sensitivity analysis, and model comparison.

Relevant analyses in this study were implemented in R v.4.2.3; the codes can be found on GitHub. All summary data used in this work are publicly available and with relevant ethical approvals.

## Results

### Preliminary screening of instrumental variables

We included 26, 26, and 18 SNPs in the MR analyses of hypertension versus AD, hypertension versus stroke, and stroke versus AD, respectively by setting the instrumental variable genome-wide correlation significance selecting p < 5e−08 and removing chain imbalance and palindromic sequences. Detailed information on specific included instrumental variables is provided in the Supplementary Material.

### MR

**Figure 2:**
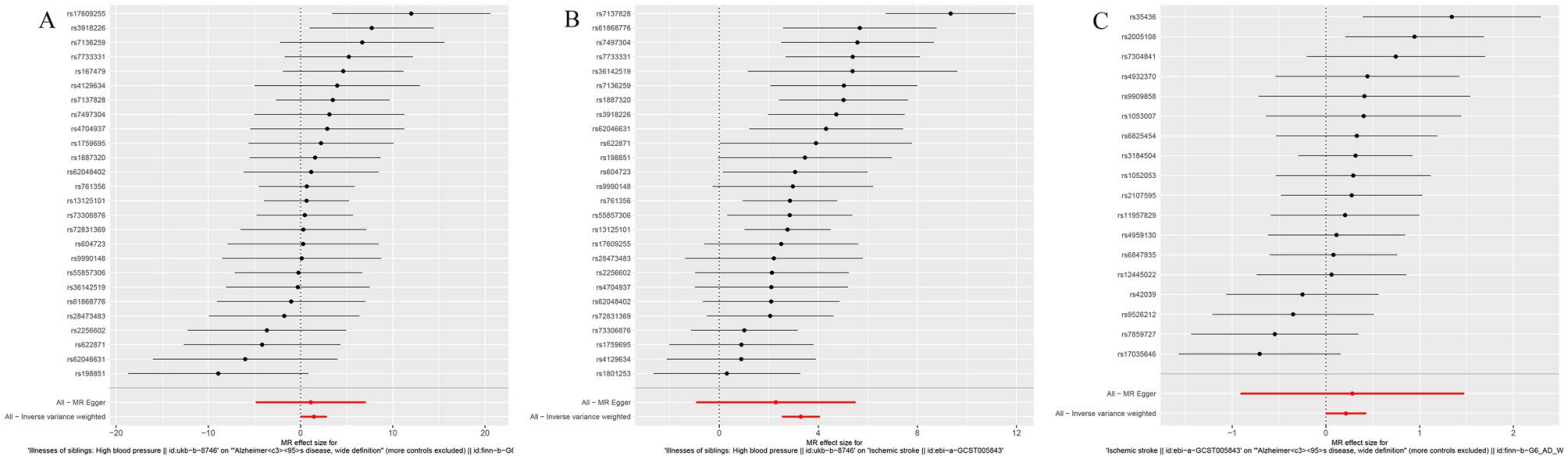
Forest map. (**A**) Hypertension and AD Forest Map, (**B**) hypertension and Stroke forest map, (**C**)stroke and AD Forest Map.

The two-sample MR analysis that we conducted in this study revealed a positive association between hypertension and AD, with stroke serving as a mediating factor. Our results further showed that hypertension is a significant risk factor for AD, with MR results of p = 0.04 and OR (95% CI) = 4.3 (1.04, 17.6) for the IVW result. Additionally, a positive correlation was observed between hypertension and stroke (IVW p = 3.8e−17, OR (95% CI) = 27.2 (12.5, 58.2)). The results are shown in the forest plot (Fig. 2A–C). Our results suggest that stroke plays a direct role in both AD and hypertension and also plays an indirect mediating role. Therefore, we regard stroke as an indirect mediating variable that contributes to hypertension leading to AD.

**Figure 3:**
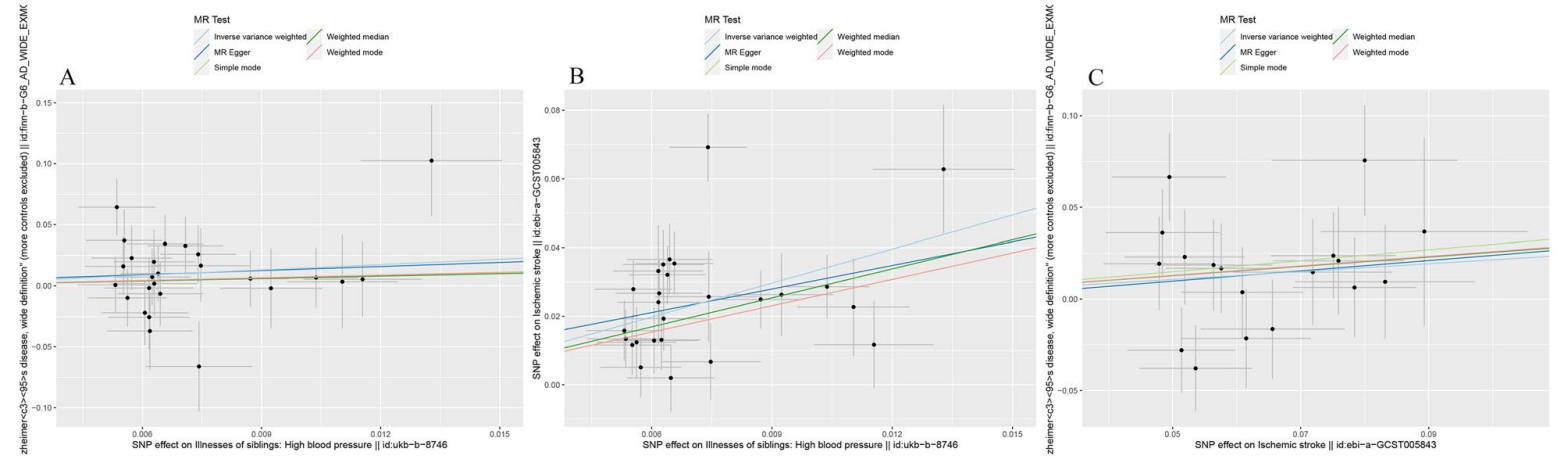
Scatter plot. (**A**) Scatter plot of hypertension and AD, (**B**) scatter plot of hypertension and stroke, (**C**) scatter plot of AD and stroke.

In MR analysis, the direct and indirect causal effects of exposure on outcome can be categorized into indirect mediators and direct mediators. In the present study, we identified stroke as an indirect mediating variable of hypertension leading to AD. Via our analysis, we showed that hypertension is a common risk factor for both AD and stroke, and stroke is also a risk factor for AD. By using a scatter plot to represent the causal positive and negative correlations in MR, we observed that the trend of the exposure-outcome relationship was stable under different analysis modes (Fig. 3A–C).

Our findings highlight the critical role of hypertension and stroke in the development and progression of AD, thereby providing important insights into the potential prevention and treatment of this debilitating disease.

**Figure 4:**
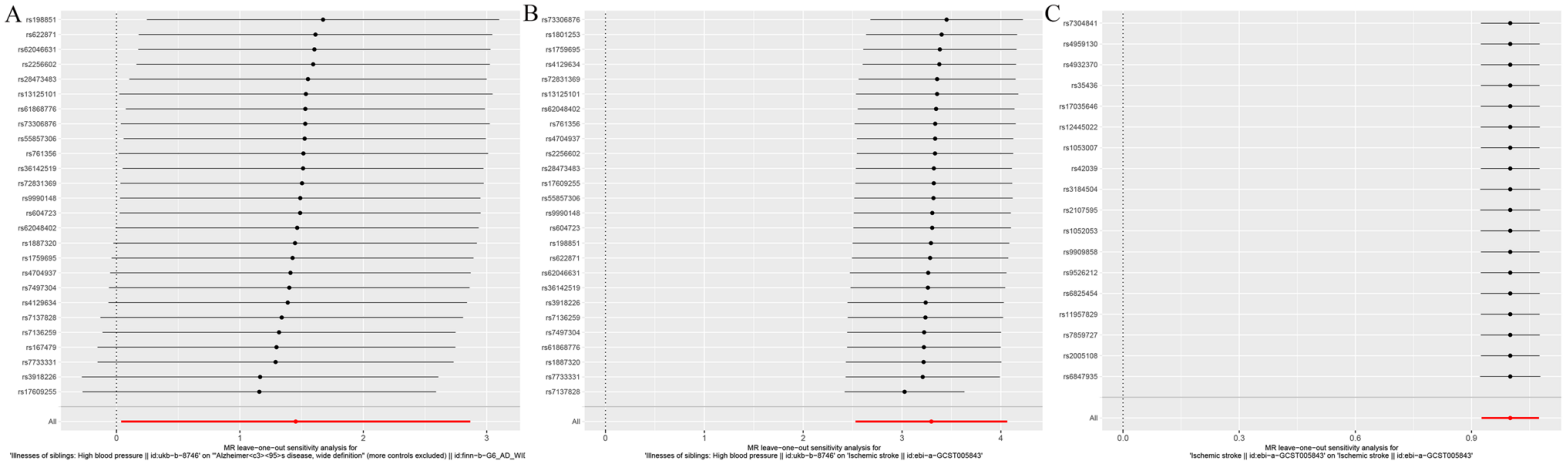
Leave-one-out plot. (**A**) Leave-one-out plot of hypertension and AD, (**B**) leave-one-out plot of hypertension and stroke, (**C**) leave-one-out plot of stroke and AD.

In this study, we conducted a sensitivity analysis using MR-PRESSO to identify and correct for outliers. We compared pre- and post-correction results to assess the impact of these outliers on the findings. Our analysis revealed Global Test P-values of 0.50 for hypertension with AD, 0.22 for stroke with AD, and 0.31 for hypertension with stroke, indicating that all three diseases were subject to outlier interference that did not significantly influence the results. We also performed rigorous sensitivity tests, including a heterogeneity test that showed Q_(MR Egger) p-values of 0.46 and 0.16 and Q_IVW values of 0.40 and 0.20 for hypertension with AD and stroke with AD, respectively. Unfortunately, the Q_(MR Egger) and Q_IVW p-values for hypertension with stroke were both less than 0.05, suggesting that the results for this relationship may have been heterogeneous. Therefore, we used the IVW random effects model, which showed consistent results. To address this issue, we applied the leave-one-out method to test data heterogeneity, and we observed a smooth trend in the leave-one-out plot (Fig. 4A–C). Finally, we performed a multiplicity test and determined p-values of 0.91 and 0.84 in the MR analysis of stroke and AD and hypertension and AD, respectively. The p-value in the MR analysis of hypertension and stroke was 0.53, indicating no multiple testing issues in our results. Mediated MR involved a two-step process and was conducted using the mediating effect formula. Multivariate mediated MR showed a mediated indirect effect value of 54.9% for stroke, indicating that approximately 55% of the risk of AD owing to hypertension may be attributed to stroke as a mediating variable. These results suggest that stroke plays an important role in the causal relationship between hypertension and AD.

## Discussion

### Key findings

In this study, we used a large consortia and summary data from GWAS to explore the causal relationship between hypertension and AD, with stroke as a mediator. We identified a significant association between hypertension and AD. The odds ratio (OR) from the MR analysis of hypertension and AD was 4.27, indicating that hypertension may contribute to a greater risk of AD. Additionally, the results from the MR analysis of hypertension and stroke and of stroke and AD also suggested that hypertension is a risk factor for stroke, and that stroke is a risk factor for AD. Furthermore, our results indicated that stroke plays an important mediating role in the process through which hypertension leads to AD.

### Potential mechanisms

Hypertension is a prevalent chronic disease that is closely associated with the increased incidence of cardiovascular diseases, stroke, and dementia, including AD^35^. There exists a complex pathophysiological relationship between hypertension and AD, particularly in the elderly population. Increasing evidence suggests an interrelation among elevated blood pressure, amyloid plaques, and neurofibrillary tangle formation in the mid-to-late stages of human brain cells^36^. Numerous clinical studies have shown the complex association between hypertension and the degree of cognitive impairment^37^. The impact of hypertension on the brain depends on the stage of the disease and the age of the patient, with the risk of dementia increasing in older adults. Additionally, clinical data from patients with AD also support this association. Patients with early-stage AD exhibit higher blood pressure values, whereas patients with late-stage AD present with severe hypertension and significant cognitive decline. These findings further support the association between hypertension and cognitive decline^38,39^. High blood pressure may contribute to the development of AD through several mechanisms. First, hypertension can lead to cerebrovascular changes. Clinical studies have shown that high blood pressure can lead to the thickening of the cerebral artery walls, reduction of cerebral blood vessel density, and narrowing of the blood vessels^40^. Prolonged exposure to these conditions can have detrimental effects on the brain’s defense mechanisms, interfere with brain function, and contribute to the development of primary dementia and cerebrovascular diseases, particularly stroke. These cerebrovascular changes can accelerate the progression of AD^41^.

Second, hypertension can also cause microvascular brain damage. Research has shown that high levels of β-amyloid (Aβ) can induce progressive multidimensional microvascular damage, which plays a crucial role in the early stages of AD^39^. Hypertension accelerates Aβ-induced microvascular damage, thereby exacerbating the severity of AD and accelerating disease progression^39^. Additionally, hypertension can lead to neuroinflammatory responses, further worsening the pathological processes of AD.

Furthermore, hypertension, Aβ deposition, and the abnormal phosphorylation of tau protein are reportedly associated^42^. Hypertension can accelerate Aβ deposition and the abnormal phosphorylation of tau protein, further promoting the development of AD^39^. Hypertension-induced cerebral small vessel inflammation and the intraneuronal hyperphosphorylation of tau protein may be key factors in this process. These pathological changes can lead to neuronal damage and cognitive decline^43^. In summary, hypertension can contribute to the development of AD through various mechanisms. Cerebrovascular changes, microvascular damage, Aβ deposition, abnormal tau protein phosphorylation, and inflammation induced by hypertension may be the key mechanisms linking hypertension and AD. Multiple clinical trials have investigated the therapeutic effects of antihypertensive drugs on AD in recent years. One study, based on a retrospective matched case–control study method, analyzed data from 122,351 participants (13,611 AD patients and 108,740 individuals without AD) who used at least one antihypertensive drug between October 1998 and April 2018. The findings of the study showed that the use of ACE inhibitors or β-blockers alone or the combination of ARBs and β-blockers could reduce the risk of AD to an extent^44^. Similar results were reported in other studies^41,45–47^. In addition, other hypertension-related diseases, such as diabetes, obesity, and hyperlipidemia, may also promote hypertension-induced AD. Hypertension, diabetes, obesity, and hyperlipidemia are common chronic metabolic diseases and known contributors to the progression of AD^48^. These metabolic diseases can increase inflammation, oxidative stress, and neuronal damage, thereby promoting the development of AD. Diabetes can increase blood glucose levels, thus increasing the risk of vascular and nerve degeneration. Obesity is often accompanied by chronic low-grade inflammation and insulin resistance, which further exacerbate the development of AD^49^. Hyperlipidemia can cause vascular damage and arteriosclerosis, thus affecting the blood supply to the brain, while increasing inflammation and oxidative stress and damaging brain neurons^50^. Therefore, preventing and controlling diabetes, obesity, and hyperlipidemia are of great significance in reducing the risk of AD^51^. Hypertension is also a major risk factor for stroke and significantly increases the incidence of stroke and mortality^52,53^. The underlying mechanisms that link hypertension to stroke include atherosclerosis, vasospasm, thrombosis, increased cerebrovascular wall permeability, and cerebrovascular inflammatory response.

Atherosclerosis, the process of plaque buildup on the inner walls of arteries, is accelerated in hypertensive individuals. Prolonged hypertension can damage the intima and induce a strong inflammatory response, thus promoting atherosclerosis^54^. This can result in the stiffening and thickening of the vessel wall, reducing the vessel's elasticity and dilation capacity, which can lead to stenosis and damage to the intima. These changes further increase the risk of thrombosis and stroke^55^. The resulting stenosis, if adequately severe, can restrict blood flow, leading to ischemic stroke^56,57^. Plaque formation on vessel walls can also cause thrombosis, which leads to ischemic stroke^58^. Additionally, hypertension induces an inflammatory response, which can increase vascular permeability and platelet aggregation, further increasing the risk of thrombosis and stroke^59^. Inflammatory responses and apoptosis in the cerebrovascular wall can also lead to vascular inflammation, further elevating the risk of stroke^60^.

In conclusion, hypertension is a major risk factor for stroke, which is influenced by atherosclerosis, vasospasm, thrombosis, increased cerebrovascular wall permeability, and cerebrovascular inflammatory responses. The connection between hypertension and AD warrants further research owing to the common mechanisms.

Based on current research, hypertension is clearly a significant risk factor for the development of AD and stroke. The mechanisms contributing to this association involve the deposition of Aβ protein, neuronal death, neurological damage, and vascular pathology. Stroke, in particular, plays a crucial role in the relationship between hypertension and AD. Therefore, preventing and controlling hypertension is of great significance in reducing the risk of AD. Maintaining a healthy lifestyle, including a balanced diet, moderate exercise, and regular check-ups, can also help prevent other metabolic diseases. With respect to antihypertensive drug therapy, combination therapy may be more effective than using a single drug. However, additional research is needed to determine the optimal treatment plan. In addition, the safety of drugs and potential adverse interactions should be assessed before devising a treatment plan.

### Comparison with other studies

Our study results are consistent with those of a previous MR study, which also identified a causal relationship between hypertension and the increased risk of AD^61^. However, in our study, we not only conducted a two-sample MR investigation on hypertension and AD but also analyzed stroke as a mediating variable. Our results show that stroke accounts for more than 50% of the mediating effect in the process by which hypertension induces AD, suggesting that hypertension may not directly cause AD, but may first trigger other conditions that lead to AD. This indicates a novel direction for mechanistic research on hypertension-induced AD and also provides novel insights into AD prevention and hypertension control.

### Advantages and limitations

This study utilized large GWAS datasets from AD, hypertension, and stroke, allowing for a systematic investigation of the causal relationships between the three. Additionally, this study is the first to use the Mendelian randomization method to analyze the mediating variables in the process of hypertension-induced AD. The various analytical methods employed in this study enhance the accuracy and reproducibility of the results. Furthermore, a series of sensitivity analyses were conducted to ensure the robustness of the findings.

Despite these advantages, it is important to acknowledge certain limitations. Firstly, the selected samples were primarily from European cohorts and did not include other populations such as Asians and Americans. This limitation may reduce the generalizability of the study results to other populations. Secondly, limitations associated with database statistics are evident. Limited statistical data, coupled with a lack of comprehensive information on the original data (e.g., inclusion/exclusion criteria, interventions), may adversely affect the accuracy of the results. Thirdly, in our study, heterogeneity was observed in the relationship between hypertension and stroke in the sensitivity analysis using the Cochran Q test. The presence of heterogeneity may be due to differences in analysis platforms, experiments, populations, and other instrumental variables. Therefore, we used an IVW random-effects model for analysis. The random-effects model is a method that can handle heterogeneity. In the random-effects model, the effect of each genetic variation is considered as a random variable rather than a fixed value. This allows for the consideration of heterogeneity between different genetic variations and assigns different weights to the estimates of different genetic variations. By using the random-effects model, the causal effects can be more accurately estimated while taking into account the influence of heterogeneity^62^.

## Conclusions

In this study, using two-sample Mendelian randomization analysis with mediation, We found that there is a positive correlation between hypertension and AD, with the risk of AD increasing as the risk of hypertension increases. Furthermore, we found that stroke plays an important mediating role in the causal relationship between hypertension and AD. Specifically, hypertension and stroke were identified as risk factors for AD, and stroke accounted for approximately 55% of the total effect of hypertension on AD.

Overall, this study provides new insights into the mechanisms by which hypertension leads to AD and highlights the significant mediating role of stroke. However, it is important to consider the limitations of this study, such as the predominantly European sample and potential database limitations. Further research is needed to validate these findings and explore other potential mediating variables in the relationship between hypertension and AD.

### Supplementary Information

Supplementary Information.

## Data Availability

The data used in the present study are all publicly available at https://gwas.mrcieu.ac.uk/.
